# Quantifying and correcting bias due to outcome dependent self-reported weights in longitudinal study of weight loss interventions

**DOI:** 10.1038/s41598-023-41853-4

**Published:** 2023-11-04

**Authors:** Jiayi Tong, Rui Duan, Ruowang Li, Chongliang Luo, Jason H. Moore, Jingsan Zhu, Gary D. Foster, Kevin G. Volpp, William S. Yancy, Pamela A. Shaw, Yong Chen

**Affiliations:** 1 Department of Biostatistics, Epidemiology and Informatics, Perelman School of Medicine, University of Pennsylvania, Philadelphia, PA 19104 USA; 2 Department of Biostatistics, Harvard T.H. Chan School of Public Health, Harvard University, Boston, MA 02115 USA; 3 Department of Computational Biomedicine, Cedars-Sinai Medical Center, Los Angeles, CA USA; 4 Division of Public Health Sciences, Department of Surgery, Washington University in St. Louis, St. Louis, MO 63110 USA; 5 Center for Health Incentives and Behavioral Economics, Perelman School of Medicine, University of Pennsylvania, Philadelphia, PA 19104 USA; 6 WW International, New York, NY 10010 USA; 7 Center for Weight and eating Disorders, Perelman School of Medicine, University of Pennsylvania, Philadelphia, PA 19104 USA; 8 Department of Medicine, Perelman School of Medicine, University of Pennsylvania, Philadelphia, PA 19104 USA; 9 Department of Medicine, Duke University, Durham, NC 27705 USA; 10 Biostatistics Division, Kaiser Permanente Washington Health Research Institute, Seattle, WA 98101 USA

**Keywords:** Statistical methods, Epidemiology

## Abstract

In response to the escalating global obesity crisis and its associated health and financial burdens, this paper presents a novel methodology for analyzing longitudinal weight loss data and assessing the effectiveness of financial incentives. Drawing from the Keep It Off trial—a three-arm randomized controlled study with 189 participants—we examined the potential impact of financial incentives on weight loss maintenance. Given that some participants choose not to weigh themselves because of small weight change or weight gains, which is a common phenomenon in many weight-loss studies, traditional methods, for example, the Generalized Estimating Equations (GEE) method tends to overestimate the effect size due to the assumption that data are missing completely at random. To address this challenge, we proposed a framework which can identify evidence of missing not at random and conduct bias correction using the estimating equation derived from pairwise composite likelihood. By analyzing the Keep It Off data, we found that the data in this trial are most likely characterized by non-random missingness. Notably, we also found that the enrollment time (i.e., duration time) would be positively associated with the weight loss maintenance after adjusting for the baseline participant characteristics (e.g., age, sex). Moreover, the lottery-based intervention was found to be more effective in weight loss maintenance compared with the direct payment intervention, though the difference was non-statistically significant. This framework's significance extends beyond weight loss research, offering a semi-parametric approach to assess missing data mechanisms and robustly explore associations between exposures (e.g., financial incentives) and key outcomes (e.g., weight loss maintenance). In essence, the proposed methodology provides a powerful toolkit for analyzing real-world longitudinal data, particularly in scenarios with data missing not at random, enriching comprehension of intricate dataset dynamics.

## Introduction

Obesity is a common epidemic disease across the whole world. During the past decades, there has been a dramatic increase in obesity. As of 2021, more than 70% of adults ages 20 and over are overweight or obese in the United States^1–6^. Increasing the incidence of diabetes, heart disease, hypertension, stroke, certain cancers, and early mortality^5–7^, obesity threatens people’s health and becomes the most significant factor to illness^8,9^. What’s more, obesity also brings a burden to health care resources and raises medical expenditures. Facing this phenomenon, it is critically needed to deploy strategies that can effectively reduce body weight and maintain weight loss.

There are some strategies for achieving weight loss that have been successfully identified; however, for people with obesity, it is more challenging to maintain long-term weight loss^10–12^. Based on a few existing studies, it is very common to observe weight regain after initial weight loss^7,12^. Factors including reduced resting metabolic rate, unregulated behavioral processes, difficulty adhering to the diet, lack competing rewards and reinforcement are all possible contributors to the difficulty in maintaining weight loss^13^. External motivation such as financial incentives can be helpful and effective at helping people achieve initial weight loss compared with the standard approaches^14^. Financial incentives have been shown to be effective in initial weight loss^14–17^. To investigate and examine the long-term effects of financial incentives on weight-loss maintenance, a study called Keep It Off was conducted.

Keep It Off study is a three-arm randomized controlled trial with two phases after initial weight loss. Participants were randomized into three groups and given different financial incentives; the three arms were (1) a lottery-based incentive, (2) a direct payment incentive and (3) a control with no financial incentive. The daily weight of participants was measured through a wireless scale at home and for those on a financial incentive arm, receipt of the incentives was reliant on attaining the goal weight. Keep If Off study provides real-world longitudinal data from 189 participants to study the relative effectiveness of lottery-based and traditional direct payment incentives on weight loss maintenance. For studies with longitudinal data, missing data are often unavoidable, and more so when participants are relied upon to provide the data themselves, whether self-reported via phone or a website, or uploaded via wireless devices as in the Keep It Off Study. Missing data can significantly threaten the results, leading to harmful effects on the validity of conclusions and decision-making.

To analyze longitudinal data, mixed-effect model^18^ and GEE (Generalized Estimating Equations)^19^ methods are commonly used methods as the standard procedure. Among these two methods, GEE is robust to the misspecification of the correlation structure of the response. Additionally, it relaxes the distribution assumption on the data and can obtain consistent estimates of the population average effect. With these features, the GEE method has been widely used in biomedical studies for longitudinal data^20–23^. On the other hand, the GEE method is less robust to non-randomly missing longitudinal data. By assuming the observation times are predefined and are the same across subjects, the GEE method provides valid results when the observations are missing completely at random (MCAR). MCAR refers to the case that the missing data are independent of both observed and unobserved variables. MCAR is the simplest case in missing data problems, but it rarely happens in practice. The GEE method can implement the inverse probability weighting approach to handle missing data even when the data are missing at random (MAR)^24^. MAR refers to when data missingness depends on observed variables alone but not the unobserved ones.

Due to the nature of the design using self-weight measurements in the Keep If Off study, there is a chance that some participants chose not to weight themselves because of potentially disappointing results, such as small weight changes or weight gains, which is a common phenomenon in many weight-loss studies and self-reported outcome studies. The missingness in this longitudinal data most likely falls into the category of missing not at random (MNAR), which refers to the case where the missingness is allowed to depend on the variables that are missing. When the data are MNAR, the GEE method will yield biased estimates because GEE has a strong assumption of independence between the observation time (i.e., self-reporting process) and the outcome of interest (i.e., weight)^25–31^.

**Figure 1:**
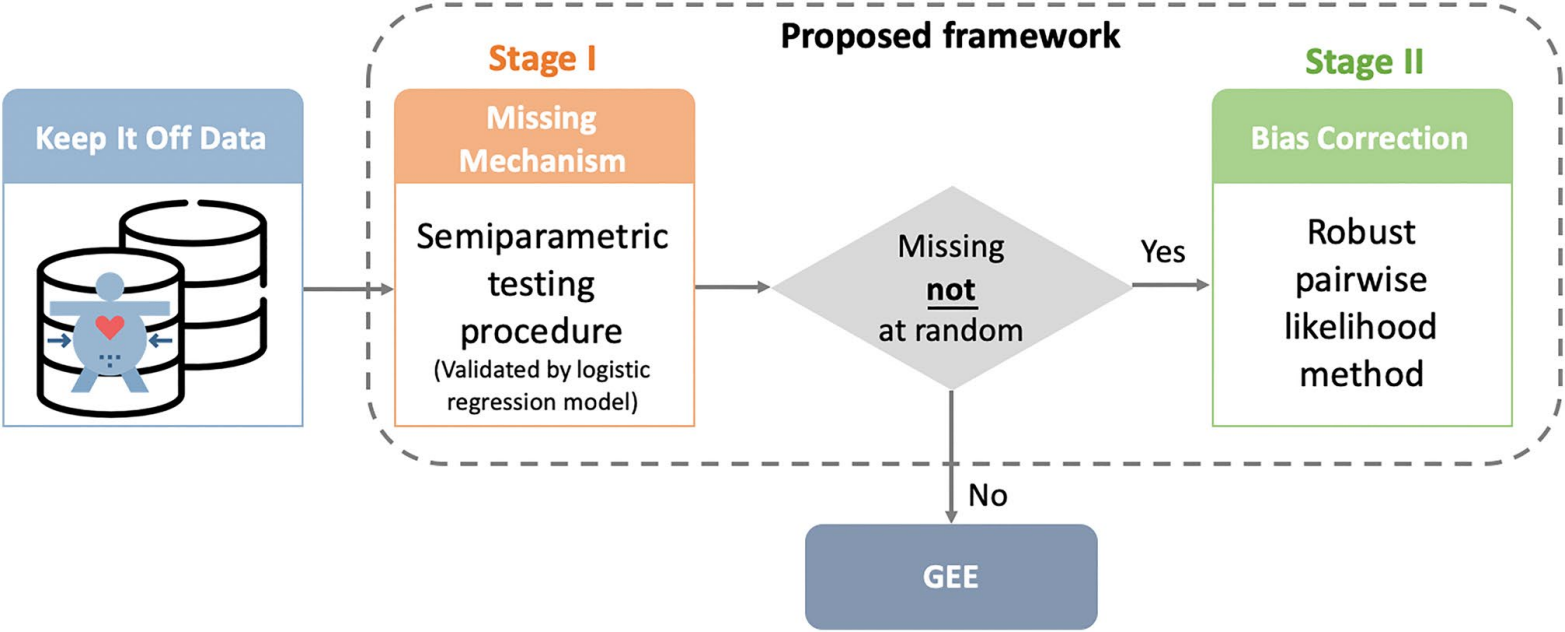
The proposed framework for the Keep It Off data analysis.

Therefore, to tackle the challenges of the informative reporting process in the real-world longitudinal data, we proposed a framework of methods with two stages in this paper (Fig. 1). In Stage I, a semiparametric testing approach^32^ was utilized to quantify the evidence of MNAR due to the self-weighing mechanism. There existed evidence indicating that the data were MNAR in the testing and validation procedures. As the data were missing not at random, the observation process was challenging to model. Thus, in Stage II, we used a pairwise likelihood method^26^, which does not require modeling the self-reporting process, to evaluate the impacts of the financial incentive on weight loss maintenance. With the proposed framework, we found that the enrollment time (i.e., days from the first day of enrollment to the weighing day, duration time) was associated with the weight loss maintenance after adjusting for the baseline participant characteristics (e.g., age, sex). There was some evidence that the participants in the groups with financial incentives (i.e., lottery group and direct payment group) would maintain weight loss better compared to the control group over time and that the lottery-based inventive was more likely to be effective for weight loss maintenance compared with the direct payment incentive.

## Results

### Keep It Off data

Keep It Off study is a three-arm, unblinded randomized controlled trial (RCT). The participants were recruited from WeightWatchers (WW), which is a global weight management program with over 4 million members and is empirically validated (ClinicalTrials.gov Identifier: NCT00702455)^33–35^. The participants aged 30–80 years old who were in stable health and had a body mass index (BMI) of 30–45 $\mathrm{kg}/{\mathrm{m}}^{2}$ before joining WW and had lost at least 11 lb before the start of the Keep It Off study to be eligible to enroll. Based on the inclusion criteria, the total number of randomized participants was 191. The participants’ baseline characteristics at the beginning of the Keep It Off study are summarized in Table 1.

**Table 1:** Available baseline characteristics of the study participants.

Characteristic	Total (n = 191)	Control (n = 39)	Direct payment (n = 75)	Lottery (n = 77)
Age, mean (SD)	49.0 (10.5)	50.1 (10.0)	48.6 (11.0)	48.8 (10.3)
Female, n (%)	175 (91.6)	36 (92.3)	69 (92.0)	70 (90.9)
Baseline BMI $(\mathrm{kg}/{\mathrm{m}}^{2})$, mean (SD)	36.7 (4.2)	36.8 (4.3)	36.7 (4.1)	36.6 (4.4)
Baseline weight (lb), mean (SD)	198.9 (32.9)	201.0 (33.1)	200.0 (34.6)	196.7 (31.5)

The Keep It Off study has two phases: an intervention phase (Phase I) and a follow-up phase (Phase II). Each phase lasted 6 months. In Phase I, the participants were randomized to get one of three interventions, including the control intervention which is daily weigh-ins and report without any incentive (referred to as control group), control intervention plus a traditional direct payment incentive (referred to as direct payment group), and control intervention plus a lottery-based incentive (referred to as lottery group). The participants who achieved their weight goals would get the lottery-based incentive or the direct payment incentive in the corresponding arm. In the study, the daily weights were collected through an Internet-enabled scale, which allowed wireless transmission to the database of weights measured daily at home. At the end of Phase I (i.e., at month 6), an *in-person milestone weigh-in* was required for all participants. This milestone weigh-in was aiming to examine if the participants reached/maintained the target weight.

In Phase II, all participants were observed without any intervention for an additional 6 months but were asked to continue weighing themselves daily as part of the ongoing study protocol. During the whole study, one of the participants became pregnant and one was diagnosed with lymphoma. Based on the inclusion criteria, these two participants are excluded and the final sample size of the participants for analysis is 189. The design and planned analysis of the Keep It Off study is detailed in the protocol paper by Shaw et al.^7^.

**Figure 2:**
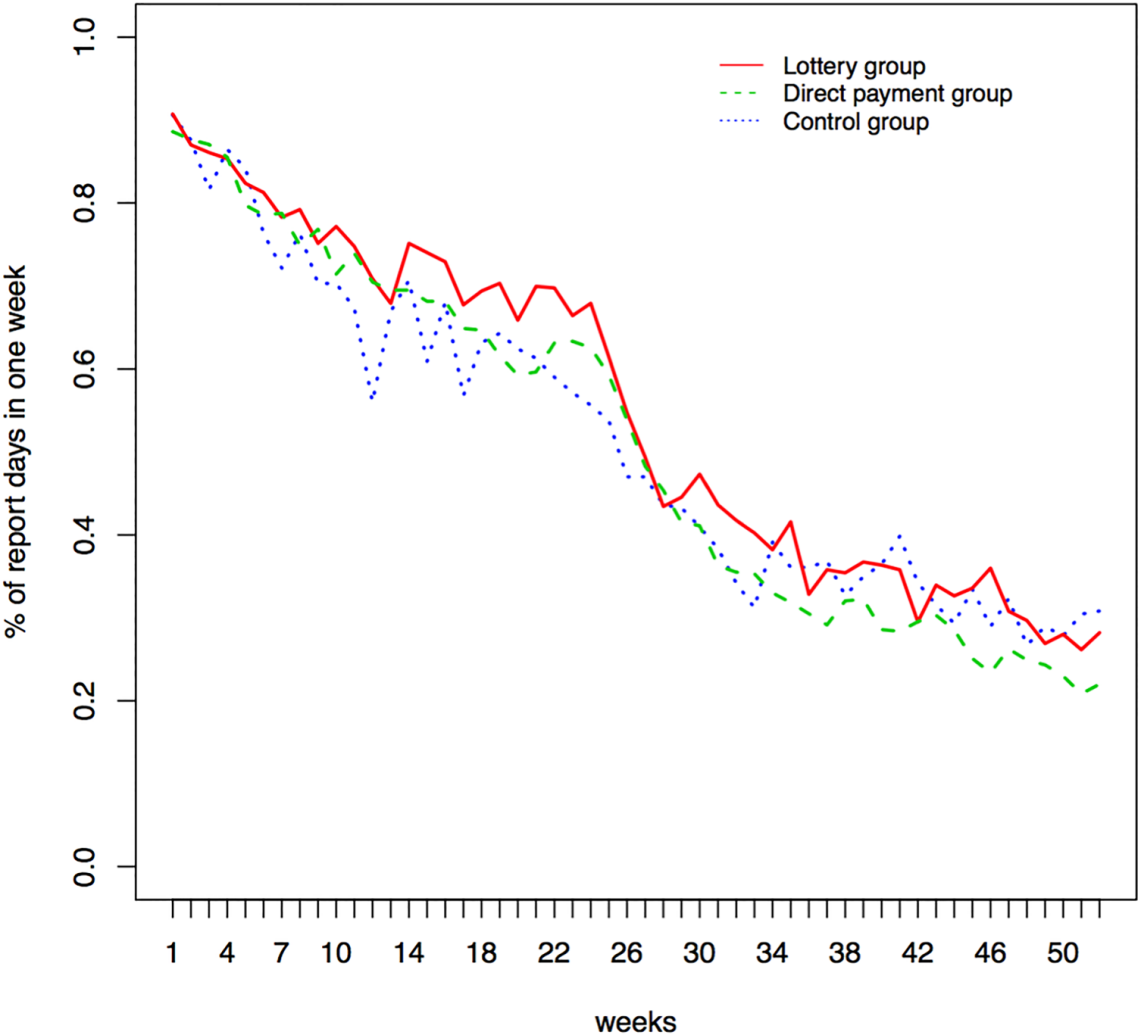
Percentage of report days in 1 week. The red line represents the lottery group; the green one represents the group with direct payment; the blue line is the control group.

With the aim of comparing effective financial incentives in the weight-loss maintenance across three groups, we first examined the patterns of reporting days as a follow-up analysis in addition to the primary analyses reported by Yancy et al.^13^. We compared the percentages of report days in one week for the three treatment groups for 12 months (i.e., 52 weeks), including both Phases I and II. Figure 2 showed the report days patterns across the treatment groups in the Keep It Off study^13^. A decreasing trend was observed through the whole study period and a big drop occurred around week 26, which was the end of Phase I (i.e., end of financial incentives). In particular, the participants weighed themselves at home and reported the weights approximately 90% of days in the first week, 75% of the days in week 10, and 55% of the days in week 26. There is a lack of evidence suggesting a difference across three groups in the patterns over time^13^.

**Figure 3:**
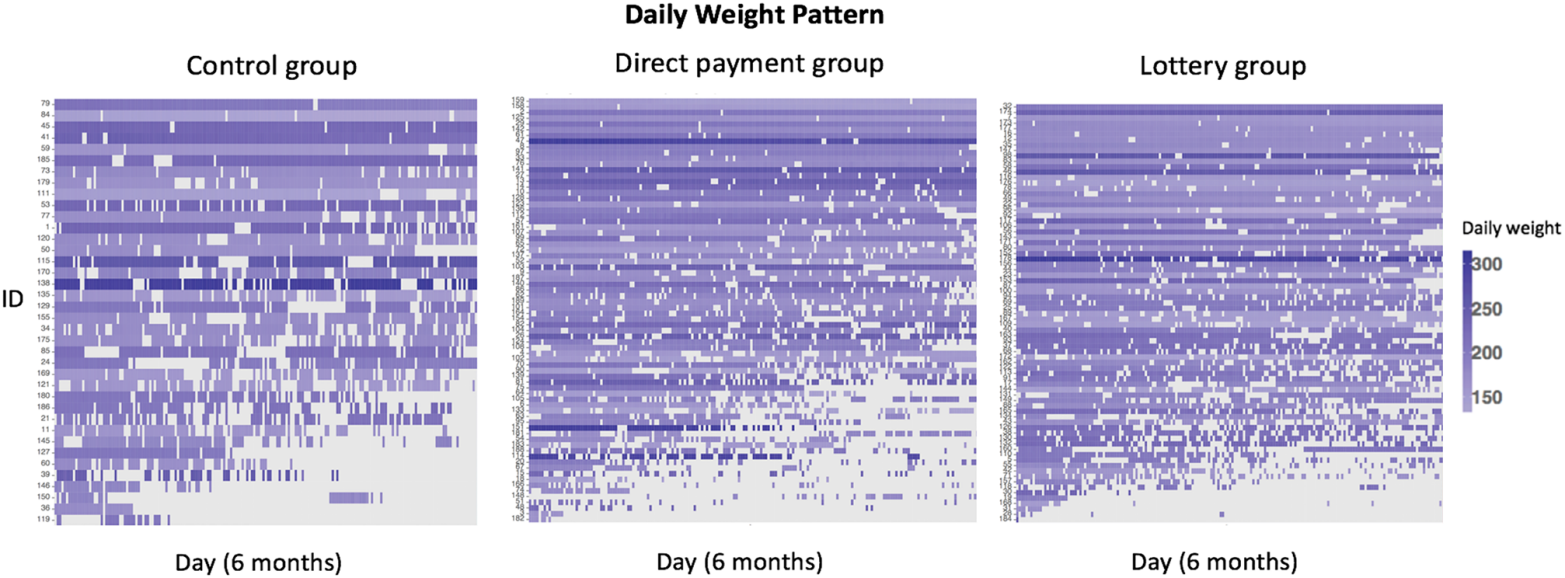
Daily weight patterns, missing percentage, and 6-month milestone weight change in the first 6 months for three groups (i.e., control group, direct payment group, lottery group). Cells in purple are the daily weights. The first column in red and green is the heat bar to show the percentage of missing daily weight. The cells in grey represent missing values.

In addition to the weekly report days pattern, we also present the reporting pattern of participants’ daily weights during Phase I in the control group (Fig. 3, left), direct payment group (Fig. 3, middle), and lottery group (Fig. 3, right). Each row was composed of the daily weights of a single participant for the first 6 months (i.e., Phase I). The purple cells were the reported daily weight, and the grey ones represented the missing values. The participants were ordered vertically from the least to the greatest percentage of missing values.

The patterns in Fig. 3 showed that the missing daily weights across all the three groups did not follow a regular pattern. Some participants reported almost every day in Phase I, but others only reported for a few days. The individual missing percentages ranged from 0.5 to 99.5%. Among the intervention groups, the control group had the most missing data (32.2%) in the first 6 months, which was larger than the missing percentages in the directly payment group (29.3%) and the lottery group (26.2%) (Table 2). This suggested that the participants with financial incentives might be more likely to maintain daily weighing and reporting.

**Table 2:** Summary of missing daily weights percentages for Phase I.

	Total (n = 191)	Control (n = 39)	Direct payment (n = 75)	Lottery (n = 77)
Phase I	28.6%	32.2%	29.3%	26.2%

Furthermore, based on the patterns in both Figs. 2 and 3, we hypothesized that the data were missing not at random. Some participants seemed to choose not to report their weights because of small weight changes or weight gains, which is a common phenomenon in many weight-loss studies and self-reported studies. Due to the use of self-weighing process in the Keep It Off study, the missing data problem is not negligible in this real-world longitudinal data. Therefore, to address the issue in missing data and investigate the effectiveness of financial incentives, we proposed a novel framework to study the missing mechanism and conduct bias correction using a robust and novel pairwise likelihood method.

### Data analysis results

We utilized our proposed framework, which composed of two stages: in Stage I, we applied a semiparametric testing approach to investigate the missing data mechanism; in Stage II, we conducted the bias correction with the estimating equation derived from pairwise composite likelihood, to analye the Keep It Off data. No standardization was performed on the variables, except for centralizing age and BMI.

In Stage I, we first applied the semiparametric testing procedure to test the missingness mechanism of the data. The test statistic is 9.12 (i.e., T = 9.12). Compared to a chi-squared distribution with 3 degrees of freedom (i.e., $m=2$ with two covariates: age and sex), the p-value equals 0.02. It indicates that there exists evidence showing the daily self-reported longitudinal data in the Keep It Off study were most likely to be *missing not at random* (*MNAR*). A discussion of the validation of the testing procedure using additional data from the Keep it Off trial is provided in Supplementary Appendix B. As there was evidence that the data were missing not random, we then conducted Stage II of the proposed framework, by applying the pairwise likelihood method. The outcome is the difference between the baseline weight and the daily self-reported weight (i.e., outcome = daily weight − baseline weight). The covariates included in the model were age at enrollment (centralized), sex, baseline BMI (centralized), time since enrollment (i.e., enrollment time, duration time), and the interaction between the time since enrollment and group indicator.

To estimate the effect sizes (i.e., regression coefficient) of the covariates, we applied both GEE method by using *gee* R package^36^ and the proposed pairwise method for comparison. The effect sizes with corresponding standard errors and the p-values of both methods are presented in Table 3. The control group was treated as the reference group in the model. The effect size of the time since enrollment on the weight change is − 0.582 (p-value = 0.048). There exists evidence showing that the longer duration time a participant remained in the study, the higher the weight loss (i.e., the duration time was positively associated with weight loss maintenance) after adjusting for the usage of the baseline participant characteristics. The lottery-based intervention (effect size = − 0.616 in Table 3) was more likely to be effective in weight loss compared with the direct payment group (effect size = − 0.512 in Table 3). The participants in the groups with financial incentives (i.e., lottery group and direct payment group) have weak evidence of maintaining weight loss better compared to the control group over time. Although the results were not statistically significant, the direction and relative magnitudes of the effect estimates are consistent with the findings from the main analysis of the Keep It Off study by Yancy Jr et al.^13^ For comparison, we also presented the marginal covariate effects estimated by the GEE method in the table. Much larger negative effects were found by the GEE method, which is likely to be overestimated due to the ignorance of the informative self-report process. The standard errors (se) estimated through the GEE method were notably larger compared to those derived from the pairwise likelihood method. This discrepancy could be attributed to the high intra-class correlation present in the data, which leads to reduced effective sample size. In the context of the weight-loss data, each patient represents a distinct cluster, and the weights associated with a single patient are naturally highly correlated.

**Table 3:** Summary of the data analysis for the associations between weight loss and the covariates.

	GEE			Pairwise likelihood		
	Effect size	SE	p-value	Effect size	SE	p-value
Sex	− 2.744	1.705	0.108	− 0.246	0.234	0.293
Baseline BMI	− 0.042	0.121	0.725	− 0.002	0.018	0.908
Age	0.087	0.047	0.060	0.013	0.007	0.050
Time since enrollment	− 7.653	2.227	0.001	− 0.582	0.295	0.048
Lottery group* time	− 4.725	2.689	0.079	− 0.616	0.342	0.072
Direct payment group* time	− 4.164	2.698	0.123	− 0.512	0.345	0.138

*For simplicity, time = time since enrollment; effect size refers to regression coefficient.

## Discussion

Here we report framework to analyze the real-world self-reported longitudinal data in the Keep It Off study. The Keep If Off study is a three-arm randomized controlled trial (RCT). The aim was to examine if participants’ weight loss maintenance can be improved by financial incentives. For the self-measured weight-loss data, the missing data problem is unavoidable. Thus, in Stage I of the proposed framework, we utilize a semi-parametric testing approach to investigate the missing mechanism of the Keep It Off data. The results showed that that the missingness of the data is most likely to be missing not at random.

For Stage II, we apply a pairwise likelihood method to evaluate the impacts of financial incentives on weight-loss maintenance. Using the conditioning technique, the pairwise likelihood method provide robust estimation of the effect of financial incentives. Without imposing parametric models on the self-reporting process, the pairwise likelihood method avoids the potential bias inherent in the GEE method under MNAR. Through the proposed framework, we show that there is a statistically significant correlation between duration time in study and weight loss. In particular, the longer the duration time, the greater the weight loss obtains.

Additionally, there is weak evidence showing that the participants in both groups with financial incentives (i.e., lottery group and direct payment group) maintain weight loss better compared to the control group over time respectively. Specifically, the lottery-based group have weak evidence of being more effective in weight loss compared with the direct payment group with control group as reference. The results are in directional agreement with those from Yancy Jr et al. for the first 6-month weight loss but not statistically significant.

As demonstrated by this study, the proposed framework is a novel framework to analyze real-world longitudinal data. In particular, the proposed framework provides a test in Stage I to examine the missing mechanism and a robust pairwise likelihood method in Stage II to investigate the association between the exposure (e.g., financial incentives) and outcome of interest (e.g., weight loss maintenance).

The proposed framework has its limitations. In Stage II, the pairwise construction of likelihood comes with the price of higher computational cost, as the algorithm involves computation of likelihood constructed by all pairs of patients within a site. To alleviate this limitation, we implemented an algorithm with R calling C, which is about 50 times faster than using the R programming language alone. The code is available on Github (https://github.com/Penncil/Keep-It-Off-Study). Secondly, the results we got were directionally consistent with Yancy Jr et al., but there was no evidence indicating the statistical significance. In summary, the proposed framework has broad applicability to other research topics where data are missing at random or completely at random, especially when the observation time process is challenging to model.

## Methods

In this section, we introduced the proposed framework to analyze the real-world self-reporting data in the Keep It Off study (ClinicalTrials.gov Identifier: NCT00702455)^35^. The framework is composed of two stages: in Stage I, we applied a semiparametric testing approach to investigate the missing data mechanism; in Stage II, we conducted the bias correction with the estimating equation derived from pairwise composite likelihood.

### Stage I: testing missing data mechanism

When analyzing the real-world healthcare data, especially the longitudinal self-reported data, the missing data problem is inevitable. In the Keep It Off study, since the participants self-weighed, there existed a large chance that participants at times chose not to use the wireless scales because of expected small weight change, failure to lose weight, or gaining weight from baseline. Under this scenario, the missingness was most likely to fall into the class of missing not at random (MNAR). Analyzing MNAR data is more challenging compared to analyzing missing at random (MAR) or missing completely at random (MCAR) data. Thus, to ensure the validity of the data analysis of Keep It Off study, we examined the missing mechanism by utilizing a semiparametric testing approach^32^.

Suppose we have a response variable $Y$ (i.e., daily weight), covariates $X$ (i.e., age, sex), and a generalized linear model, where the conditional distribution of $Y$ given covariates $X$ belongs to the exponential dispersion family:

1 $$p(y\mid x;\theta )=\mathrm{exp}\left\{\frac{y\eta -b\left(\eta \right)}{\lambda }+c\left(y;\lambda \right)\right\}, h\{\mu (\eta )\}=\alpha +{x}^{T}{\beta }_{1}$$

where $\mu =E(Y\mid X)$, is related to covariates through a link function $h(\cdot )$, $\eta$ is the natural parameter, $\lambda$ is the dispersion parameter, $\mu (\eta )={b}^{\mathrm{^{\prime}}}(\eta )$ by the property of the exponential family and ${\varvec{\beta}}={\left(\alpha ,{\beta }_{1}\right)}^{T}$ are regression coefficients of interest.

We proposed to identify two parameter estimators of the regression coefficient. Both estimators are valid when the data is missing at random, while only one of them is valid otherwise. The first estimator of ${\varvec{\beta}}$ is denoted as $\widehat{{\varvec{\beta}}}$**,** which is obtained by solving the estimating equation derived from the likelihood function using the probability density function (Eq. (1)) of exponential family. The second estimator is denoted as $\widetilde{{\varvec{\beta}}}$, which is obtained using a semiparametric pseudo-likelihood method^37^.

Our test of missingness is based on the discrepancy between the two estimators of ${\varvec{\beta}}$. The test statistics, $T$, can be written as:

2 $$T=n(\widetilde{{\varvec{\beta}}}-\widehat{{\varvec{\beta}}}{)}^{T}{\widehat{W}}^{-1}(\tilde{{\varvec{\beta}}}-\widehat{{\varvec{\beta}}})$$

where $n$ is the sample size and $\widehat{W}$ is the weighting matrix which can be estimated through the influence functions of estimators $\widehat{{\varvec{\beta}}}$ and $\tilde{{\varvec{\beta}}}$. The details of derivation of two estimators and the weighting matrix are provided in the Supplementary Appendix A. As $n\to \infty$, the test statistic T converges weakly to ${\upchi }^{2}$ with $m+1$ degree of freedom, where $m$ is the dimension of covariate $X$. It is suggested that the data is more likely to be missing not at random when the test statistic $T$ takes large values.

### Stage II: analyzing missing not at random data

For the Keep It Off study, the data are most likely to be missing not at random as we have shown. Thus, alternative methods are critically needed for the outcome-dependent longitudinal data. An innovative *pairwise likelihood method* was proposed in 2015^26^. The pairwise conditional scheme was utilized to form a composite conditional likelihood. This method provides an alternative estimating procedure for the investigation of the marginal covariate effects on the repeated measure in longitudinal weight loss data. The novelty of this method is that it does not require modeling the self-reported time process, which is challenging when the data is missing not at random. This key feature of this pairwise likelihood method brings robustness to the statistical inference and corrects the potential bias induced by the outcome-dependent weight loss data.

Suppose there are two observations from a pair of independent participants: the $j$-th observation of participant $i$ and the $j{\prime}$-th observation of participant $i{\prime}$. Let ${y}_{ij}$ denote daily weight response at the $j$-th time point of participant $i$ and ${y}_{i{\prime}j{\prime}}$ denote the daily weight response at the $j{\prime}$-th time point of participant $i{\prime}$. For one of the observations (${y}_{ij}$), the proportional density function^26,38^ given the vector of covariates ${\mathbf{x}}_{{\varvec{i}}{\varvec{j}}}$ can be written as:

3 $$f\left( {y_{ij} \left| {{\mathbf{x}}_{{{\mathbf{ij}}}} ;{\varvec{\beta}}} \right.} \right) = \frac{{{\text{exp}}\left( {{\varvec{\beta}}^{T} {\mathbf{x}}_{{{\mathbf{ij}}}} y_{ij} } \right)g\left( {y_{ij} } \right)}}{{\smallint {\text{exp}}\left( {{\varvec{\beta}}^{T} {\mathbf{x}}_{{{\mathbf{ij}}}} y_{ij} } \right)dG\left( {y_{ij} } \right)}}$$

where $g$(.) is the distribution of the response variable and $G(.)$ is the cumulative distribution, which represent the probability structure of the observation time process. To focus on the estimation of parameter of interest ${\varvec{\beta}}$, the specification of the distribution function is not necessary. Thus, the nuisance distribution function can be cancelled out through a conditioning technique. The conditional density of the responses at $j$-th time point of participant $i$ and at the $j{\prime}$-th time point of participant $i{\prime}$, (${y}_{ij},\boldsymbol{ }\boldsymbol{ }{y}_{{i}^{\mathrm{^{\prime}}}{j}^{\mathrm{^{\prime}}}}$), given the order statistics ${y}^{\left(1\right)}=\mathrm{min}({y}_{ij}, {y}_{{i}^{\mathrm{^{\prime}}}{j}^{\mathrm{^{\prime}}}})$ and ${y}^{\left(2\right)}=\mathrm{max}({y}_{ij}, {y}_{{i}^{\mathrm{^{\prime}}}{j}^{\mathrm{^{\prime}}}})$, can be calculated as:

4 $$f(y_{ij} ,\user2{ }y_{{i^{\prime}j^{\prime}}} \left| {y^{\left( 1 \right)} , y^{\left( 2 \right)} , {\mathbf{x}}_{{{\mathbf{ij}}}} , {\mathbf{x}}_{{{\mathbf{i^{\prime}j^{\prime}}}}} } \right.) = \frac{{f\left( {y_{ij} \left| {{\mathbf{x}}_{{{\mathbf{ij}}}} } \right.;{\varvec{\beta}}} \right)f\left( {y_{{i^{\prime}j^{\prime}}} \left| {{\mathbf{x}}_{{{\mathbf{i^{\prime}j^{\prime}}}}} } \right.;{\varvec{\beta}}} \right) }}{{f\left( {y_{ij} \left| {{\mathbf{x}}_{{{\mathbf{ij}}}} } \right.;{\varvec{\beta}}} \right)f\left( {y_{{i^{\prime}j^{\prime}}} \left| {{\mathbf{x}}_{{{\mathbf{i^{\prime}j^{\prime}}}}} } \right.;{\varvec{\beta}}} \right) + f\left( {y_{ij} \left| {{\mathbf{x}}_{{{\mathbf{i^{\prime}j^{\prime}}}}} } \right.;{\varvec{\beta}}} \right)f\left( {y_{{i^{\prime}j^{\prime}}} \left| {{\mathbf{x}}_{{{\mathbf{ij}}}} } \right.;{\varvec{\beta}}} \right)}}$$

where $\mathbf{x}$ is the vector of the covariates (e.g., baseline weight, BMI, age, time, etc.), and ${\varvec{\beta}}$ is the vector parameters of interest. Through conditioning on the order statistics, the probability structures of the observation time process (i.e., $g({y}_{ij})$) were eliminated in the density function. For this procedure, the specification of the observation time process is not required.

For each possible pair of participants, we can calculate the above conditional density. By multiplying these densities together, the following pairwise likelihood function for all observations can be obtained:

5 $$L({\varvec{\beta}})=\prod_{i<{i}{\prime}}\prod_{j=1}^{{K}_{i}}\prod_{{j}{\prime}=1}^{{K}_{i{\prime} }}[{\{1+\frac{f({y}_{ij}\mid {\mathbf{x}}_{{\mathbf{i}}^{\mathbf{^{\prime}}}{\mathbf{j}}^{\mathrm{^{\prime}}}};{\varvec{\beta}})f({y}_{{i}^{\mathrm{^{\prime}}}{j}^{\mathrm{^{\prime}}}}\mid {\mathbf{x}}_{\mathbf{i}\mathbf{j}};{\varvec{\beta}})}{f({y}_{ij}\mid {\mathbf{x}}_{\mathbf{i}\mathbf{j}};{\varvec{\beta}})f({y}_{{i}^{\mathrm{^{\prime}}}{j}^{\mathrm{^{\prime}}}}\mid {\mathbf{x}}_{{\mathbf{i}}^{\mathbf{^{\prime}}}{\mathbf{j}}^{\mathbf{^{\prime}}}};{\varvec{\beta}})}\}}^{-1}]$$

where ${K}_{i}$ is the number of total observations of participant $i$ and ${K}_{i{\prime}}$ is the number of total observations of participant $i{\prime}$. With this pairwise conditioning approach, the missing-not-at-random mechanism, i.e. the observation of y depending on x, $g({y}_{ij})$, is canceled in Eq. (5). In other words, the missingness pattern of the dependent variable does not affect the estimation of the parameter of interest. The final pairwise estimator is $\widehat{{\varvec{\beta}}}=\mathrm{argmax L}({\varvec{\beta}})$, which is the marginal covariate effects on the outcome.

The performance of this novel pairwise likelihood method in analyzing longitudinal data has been validated through extensive simulation studies and empirical data application in Chen et al.^26^. The key assumption of this method is that the probability of observing the response variable can be expressed as a product of functions involving the response variable and covariates, which is Eq. (2.2) in Chen et al.^26^. Their simulation studies indicate a degree of robustness in cases where this assumption does not meet. In the context omodel misclassification, Luo and Tsai^38^ emphasize the robustness of the proportional density ratio. This robustness underscores the method's stability and flexibility, ensuring its reliability in real-world scenarios where model assumptions might not hold. By incorporating the pairwise conditioning technique, our method maintains the effectiveness in analyzing the weight loss data.

**Figure 4:**
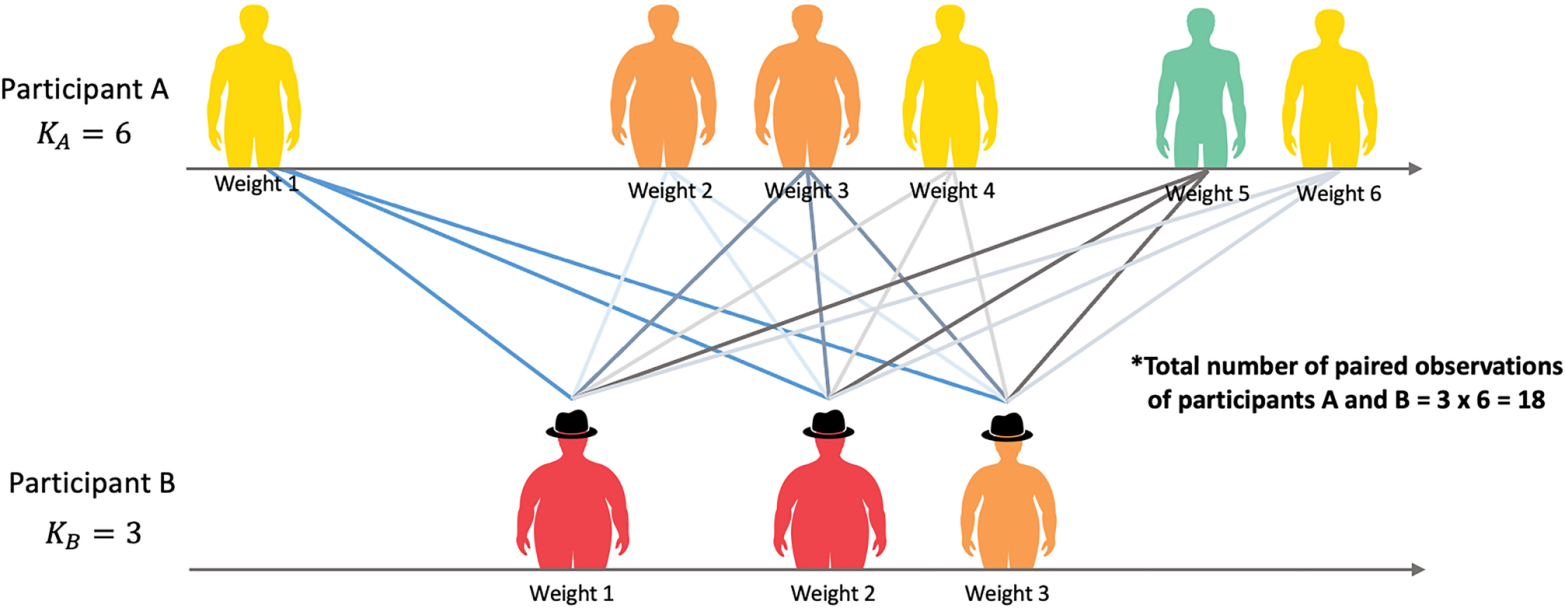
Illustration of the pairwise likelihood method idea in the proposed framework Stage II by using a pair of participants as example. K is the total number of observations for each participant.

In Fig. 4, we graphically illustrated the pairwise idea by using a pair of participants as an example. Participant A has 3 observations and participant B has 6 observations over time. The total number of pairs of observations between participants A and B is 18. With this idea, the pairs of observations of both participants were multiplied together to get the likelihood function as Eq. (5).

All methods were carried out in accordance with relevant guidelines and regulations. We confirmed all experimental protocols were approved. The use of human subjects was approved by the University of Pennsylvania Institute Review Board (IRB) under the protocol number 816917. Waivers of Informed Consent were granted by the Institutional Review Board of the University of Pennsylvania.

### Supplementary information

Supplementary Information.

## Data Availability

The data underlying this article cannot be shared publicly due to patient privacy concerns. The data will be shared on reasonable request to the corresponding author.

## Code availability

The R code is available at https://github.com/Penncil/Keep-It-Off-Study. The analyses were conducted using R version 4.2.1.
