# Publisher Correction: Quantifying and correcting bias due to outcome dependent self-reported weights in longitudinal study of weight loss interventions

**DOI:** 10.1038/s41598-023-49737-3

**Published:** 2023-12-18

**Authors:** Jiayi Tong, Rui Duan, Ruowang Li, Chongliang Luo, Jason H. Moore, Jingsan Zhu, Gary D. Foster, Kevin G. Volpp, William S. Yancy, Pamela A. Shaw, Yong Chen

**Affiliations:** 1 Department of Biostatistics, Epidemiology and Informatics, Perelman School of Medicine, University of Pennsylvania, Philadelphia, PA 19104 USA; 2 Department of Biostatistics, Harvard T.H. Chan School of Public Health, Harvard University, Boston, MA 02115 USA; 3 Department of Computational Biomedicine, Cedars-Sinai Medical Center, Los Angeles, CA USA; 4 Division of Public Health Sciences, Department of Surgery, Washington University in St. Louis, St. Louis, MO 63110 USA; 5 Center for Health Incentives and Behavioral Economics, Perelman School of Medicine, University of Pennsylvania, Philadelphia, PA 19104 USA; 6 WW International, New York, NY 10010 USA; 7 Center for Weight and eating Disorders, Perelman School of Medicine, University of Pennsylvania, Philadelphia, PA 19104 USA; 8 Department of Medicine, Perelman School of Medicine, University of Pennsylvania, Philadelphia, PA 19104 USA; 9 Department of Medicine, Duke University, Durham, NC 27705 USA; 10 Biostatistics Division, Kaiser Permanente Washington Health Research Institute, Seattle, WA 98101 USA

Correction to: *Scientific Reports* 10.1038/s41598-023-41853-4, published online 04 November 2023

The original version of this Article contained an error in the name of author William S. Yancy Jr., where the name suffix ‘Jr.’ was inadvertently omitted.

The original Article has been corrected.

