# Supplementary material for: Quantifying and correcting bias due to outcome dependent self-reported weights in longitudinal study of weight loss interventions
Source: Sci Rep. 2023 Nov 4;13:19078. doi: 10.1038/s41598-023-41853-4 (PMC10625563; doi:10.1038/s41598-023-41853-4)
Supplement: Supplementary file 1 — Supplementary Information. [file 41598_2023_41853_MOESM1_ESM.docx]

Supplementary Appendix to “Quantifying and correcting bias due to outcome dependent self-reported weights in longitudinal study of weight loss interventions”

Table of Contents

[A. Supplemental Methods 2](#_Toc144805387)

[The details of derivation of the estimators in testing missing data mechanism 2](#_Toc144805388)

[B. Supplemental Results 4](#_Toc144805389)

[The testing procedure using data from the Keep it Off trial. 4](#_Toc144805390)

[References 5](#_Toc144805391)

# A. Supplemental Methods

## The details of derivation of the estimators in testing missing data mechanism

Suppose we have a response variable $Y$ (i.e., daily weight), covariates $X$ (i.e., age, sex), and a generalized linear model, where the conditional distribution of $Y$ given covariates $X$ belongs to the exponential dispersion family:

$p(y\mid x;\theta)=exp\left\{ \frac{y\eta-b\left( \eta\right)}{\lambda}+c\left( y;\lambda\right) \right\}, h\{\mu(\eta)\}=\alpha+x^{T}\beta_{1}$ (1)

where $\mu=E(Y\mid X)$, is related to covariates through a link function $h(\cdot)$, $\eta$ is the natural parameter, $\lambda$ is the dispersion parameter, $\mu(\eta)=b^{'}(\eta)$ by the property of the exponential family and $\boldsymbol{\beta}=\left( \alpha,\beta_{1} \right)^{T}$ are regression coefficients of interest. Two two parameter estimators of the regression coefficient are proposed [1].

The first estimator of $\boldsymbol{\beta}$ is denoted as $\hat{\boldsymbol{\beta}}$, which is an estimator under missing at random. An inverse probability weighting method is used to obtain this estimator. When the true propensity $\pi_{0}(x)=P(R=1\mid X=x)$ is known, the inverse probability weighted likelihood score equation is written as as

$g_{n}(\boldsymbol{\beta})=\frac{1}{n}\sum_{i=1}^{n} \frac{r_{i}}{\pi_{0}\left( x_{i} \right)}S\left( y_{i},x_{i};\boldsymbol{\beta} \right),$ (2)

where $S(Y,X;\beta)=\nabla_{\beta}logp(Y\mid X;\beta)$. A two-stage method is used to obtain a plug-in estimating equation:

$\hat{g}_{n}(\boldsymbol{\beta})=\frac{1}{n}\sum_{i=1}^{n} \frac{r_{i}}{\hat{\pi}_{b}\left( x_{i} \right)}S\left( y_{i},x_{i};\boldsymbol{\beta} \right)=0$, (3)

where more details of $\hat{\pi}_{b}(.)$ are available in Duan et al. (2020). The first estimator solves the estimating equation (3), resulting in:

$$n^{1/2}\left( \hat{\boldsymbol{\beta}}\mathbf{-}\boldsymbol{\beta}_{\mathbf{0}} \right)\to N(0,\boldsymbol{\Sigma}),\text{ with }\boldsymbol{\Sigma}=E\left( \psi_{i}\psi_{i}^{T} \right)$$

as $n\to\infty$, where $\psi_{i}$ is an influence function of $\hat{\boldsymbol{\beta}}$**,** calculated as

$$\psi_{i}=\frac{r_{i}}{\pi_{0}\left( u_{i} \right)}E\left[ -\nabla_{\beta}S(Y,X;\boldsymbol{\beta}) \right]^{-1}S\left( y_{i},x_{i};\boldsymbol{\beta} \right).$$

The second estimator of $\boldsymbol{\beta}$ is denoted as $\tilde{\boldsymbol{\beta}}$, which is obtained using a semiparametric pseudo-likelihood method, resulting in:

$$n^{1/2}\left( \tilde{\boldsymbol{\beta}}\mathbf{-}\boldsymbol{\beta}_{\mathbf{0}} \right)\to N(0,\boldsymbol{\Omega}),\text{ with }\boldsymbol{\Omega}=E\left( \phi_{i}\phi_{i}^{T} \right)$$

as $n\to\infty$, where $\phi_{i}$ is the influence function of $\tilde{\boldsymbol{\beta}}$, calculated as

$\phi_{i}=-E\left\{ \nabla_{\beta\beta}H\left( \boldsymbol{\beta}_{\mathbf{0}},F_{0} \right) \right\}^{-1}\left\{ \nabla_{\beta}H_{i}\left( \boldsymbol{\beta}_{\mathbf{0}},F_{0} \right)+\delta_{i} \right\}$,

where $H(.)$ is the semiparametric log-pseudolikelihood function defined in Duan et al. (2020) Equation (2.5) and $F_{0}$ is the cumulative distribution function of $f(x)$. For more details, please refer to Duan et al. (2020).

With the two estimators above, the test on missingness is conducted based on the discrepancy between the two estimators of $\boldsymbol{\beta}$, as shown in the main manuscript.

# B. Supplemental Results

## **The testing procedure using data from the Keep it Off trial.**

In the manuscript, we proposed a two-stage framework. In the first stage, we investigate the missing data mechanism of the Keep It Off data. The primary outcome in the data is the participant’s weight at the in-person milestone weigh-in at the end of Phase I (i.e., at month 6).

We fit a linear regression model with the outcome as the 6-month milestone weigh-in weight and the covariates including baseline BMI, sex, age, time since enrollment, lottery group * time, and direct payment group * time, there exists evidence showing that all variables, except age (p-value = 0.36), are significantly associated with the milestone outcome with p-value < 0.01.

Then, we fit the logistic regression model with the missing indicator R as the outcome, and variables including 6-month milestone weight, baseline BMI, sex, age, time since enrollment, lottery group * time, and direct payment group * time. By conditioning on the variables, there exists evidence showing the 6-month milestone weight is significantly associated with the missing indicator with p-value < 0.01, suggesting the data are not missing at random, which is consistent with the conclusion using the testing procedure shown in the main manuscript.

# References

1 Duan R, Liang CJ, Shaw P, *et al.* Missing at random or not: a semiparametric testing approach. *arXiv preprint arXiv:200311181* 2020.
